# Alcohol’s Impact on the Fetus

**DOI:** 10.3390/nu13103452

**Published:** 2021-09-29

**Authors:** Svetlana Popova, Danijela Dozet, Kevin Shield, Jürgen Rehm, Larry Burd

**Affiliations:** 1 Institute for Mental Health Policy Research, Centre for Addiction and Mental Health, 33 Ursula Franklin Street, Toronto, ON M5S 2S1, Canada; danijela.dozet@camh.ca (D.D.); kevin.shield@camh.ca (K.S.); jtrehm@gmail.com (J.R.); 2 Dalla Lana School of Public Health, University of Toronto, 155 College Street, Toronto, ON M5T 3M7, Canada; 3 Factor-Inwentash Faculty of Social Work, University of Toronto, 246 Bloor Street W, Toronto, ON M5S 1V4, Canada; 4 Institute of Medical Science, Faculty of Medicine, Medical Sciences Building, University of Toronto, 1 King’s College Circle, Toronto, ON M5S 1A8, Canada; 5 Institute of Clinical Psychology and Psychotherapy & Center of Clinical Epidemiology and Longitudinal Studies, Technische Universität Dresden, Chemnitzer Street 46, 01187 Dresden, Germany; 6 Department of Psychiatry, University of Toronto, 250 College Street, Toronto, ON M5T 1R8, Canada; 7 Department of International Health Projects, Institute for Leadership and Health Management, I.M. Sechenov First Moscow State Medical University, Trubetskaya Street, 8, b. 2, 119992 Moscow, Russia; 8 Department of Pediatrics, University of North Dakota School of Medicine and Health Sciences, 1301 N Columbia Rd., Grand Forks, ND 58202, USA; larry.burd@ndus.edu

**Keywords:** alcohol, fetal, fetal alcohol spectrum disorder, morbidity, mortality, pregnancy

## Abstract

Background: Alcohol is a teratogen and prenatal exposure may adversely impact the developing fetus, increasing risk for negative outcomes, including Fetal Alcohol Spectrum Disorder (FASD). Global trends of increasing alcohol use among women of childbearing age due to economic development, changing gender roles, increased availability of alcohol, peer pressure and social acceptability of women’s alcohol use may put an increasing number of pregnancies at risk for prenatal alcohol exposure (PAE). This risk has been exacerbated by the ongoing COVID-19 pandemic in some countries. Method: This literature review presents an overview on the epidemiology of alcohol use among childbearing age and pregnant women and FASD by World Health Organization regions; impact of PAE on fetal health, including FASD; associated comorbidities; and social outcomes. Results/Conclusion: The impact of alcohol on fetal health and social outcomes later in life is enormous, placing a huge economic burden on countries. Prevention of prenatal alcohol exposure and early identification of affected individuals should be a global public health priority.

## 1. Introduction

Alcohol consumption can harm not only the person consuming alcohol, but also to their family, friends and community. Alcohol use during pregnancy is one of the examples of “harm to others”—to the developing fetus. Alcohol has been a well-established teratogen for many years and may adversely impact the developing fetus, increasing risk for many adverse outcomes, including Fetal Alcohol Spectrum Disorder (FASD).

Alcohol use is increasing at an alarming rate among women globally, particularly among women of childbearing age (15–49 years) and pregnant women [1,2,3,4,5]. Alcohol consumption has increased in terms of the prevalence of current drinkers [4], and in the volume and frequency of drinking among drinkers [5], as well as the lowering of the age of initiation for alcohol use [3]. Though a global assessment of alcohol use among pregnant women during the pandemic has yet to be conducted, there is some evidence to suggest that the COVID-19 pandemic has been associated with increased alcohol use among women of childbearing age in some countries [6,7,8]. Higher than usual rates of alcohol use, combined with unplanned pregnancies, places many pregnancies at increased risk of alcohol exposure and therefore, many infants at risk of harm [9]. Changes to substance use during the pandemic may be mediated by mental health status; for example, pregnant women’s psychological distress in the United States was predictive of the number of substances they used [10], whereas a Canadian study found no such association [11]. This article will overview epidemiology of alcohol use among childbearing and pregnant women, FASD in general and special sub-populations as well as the impact of alcohol on the fetus.

### 1.1. Alcohol Use among Childbearing-Aged Women

Based on data from the World Health Organization’s (WHO) 2018 Global Information System on Alcohol and Health [4], global adult per capita consumption of alcohol (APC) among women of childbearing age was estimated to be 2.3 L in 2016. Worldwide, APC among women of childbearing age varies, ranging from as low as 0.1 L in the Eastern Mediterranean Region to as high as 4.6 L in the European Region. Countries with high-income economies reported the greatest APC (4.5 L), followed by upper-middle-income economies (2.7 L), lower-middle-income economies (1.5 L), and low-income economies (1.2 L) (see Table 1).

Globally, in 2016, 32.1% of women of childbearing age consumed alcohol. The prevalence of current drinking among childbearing-aged women was lowest in the Eastern Mediterranean Region (1.3%) and highest in the European Region (53.9%) [4].

Worldwide, approximately 8.7% of women of childbearing age engaged in heavy episodic drinking (HED), defined as consuming at least 60 g of pure alcohol on at least one occasion in the past 30 days [4]. The definition of what constitutes a standard drink varies: in the United States, a standard drink contains 14 g of pure alcohol, but in Europe, a standard drink contains approximately 10 g of alcohol.

Among women of childbearing age who drink, the prevalence of HED was lowest in the Eastern Mediterranean Region (0.1%) and highest in the European Region (18.7%). Among childbearing-aged women who drink, the prevalence of HED was highest in countries with high-income economies (60.7% and 17.3%, respectively), followed by upper-middle-income economies (37.6% and 10.4%, respectively), lower-middle-income economies (20.6% and 5.0%, respectively), and low-income economies (17.4% and 5.5%, respectively).

The ratio of men’s APC to women’s APC in 2016 was estimated to be 4.6 globally; highest in the Eastern Mediterranean Region (10.8) and lowest in the European Region (3.8). Assuming a prospective trend of convergence of gendered alcohol use [13], consumption by women is expected to continue to increase in those regions where currently there exists a large discrepancy in the amount of alcohol consumed by men versus women.

### 1.2. Alcohol Use during Pregnancy

Many countries provide information to healthcare practitioners and the public regarding the detrimental effects of alcohol consumption during pregnancy, often in the form of clinical guidelines. Examples include guidelines produced by Australia [14], Denmark [15], Canada [16], France [17], the United States [18], and the WHO’s 2014 guidelines for the management of substance use during pregnancy [19]. Currently, no level of alcohol exposure during pregnancy is known to be safe [20], as even relatively low levels of alcohol use can substantially increase the risk of FASD [21]. Despite these public health efforts, approximately 10% of women worldwide continue to consume alcohol during pregnancy [22,23].

The WHO European Region (EUR) was estimated to have the highest prevalence of alcohol use during pregnancy (25.2%) [22] (see Table 2). This is not surprising; according to the latest Global Status Report on Alcohol and Health [4], the European Region ranked highest in nearly all major alcohol indicators, including prevalence of consumption, levels of consumption, rates of alcohol use, HED, and Alcohol Use Disorders (AUDs).

Additionally, the five countries with the highest prevalence of alcohol use among pregnant women were in the European Region: Ireland (60.4%), Belarus (46.6%), Denmark (45.8%), the United Kingdom (41.3%), and Russia (36.5%) [22,23]. Among these five countries alone, there are an annual 902,180 prenatally alcohol-exposed births, 69,395 of which have FASD. In total, there are approximately 1,249,110 cases with FASD among those aged 0–18 years in these five countries alone (see Table 3).

The lowest prevalence of alcohol use during pregnancy was estimated to be in the Eastern-Mediterranean Region (50 times lower than the global average) followed by the South-East Asia Region (five times lower than the global average); in these regions, a large proportion of the population abstains from alcohol use, especially within the female population [19].

In addition to high rates of alcohol consumption during pregnancy, in 40% of the countries examined, over one-quarter of pregnant women who consumed alcohol engaged in binge drinking [23,24]. This is alarming, as binge drinking is the most detrimental pattern of drinking during pregnancy and increases the risk of FASD. The highest prevalence of binge drinking during pregnancy was estimated to be in the African Region (3.1%), while the lowest prevalence of binge drinking during pregnancy was estimated to be in the Western-Pacific Region (1.8%) (see Table 2).

Among women who drank any amount of alcohol during pregnancy, the proportion who engaged in binge drinking was estimated to range from 10.7% in the European Region to 31.0% in the African Region [23]. The five countries with the highest estimated prevalence of binge drinking during pregnancy were Paraguay (13.9%), Moldova (10.6%), Ireland (10.5%), Lithuania (10.5%), and the Czech Republic (9.4%) [23].

These estimates are representative of the general populations of the respective countries; however, prevalence rates of alcohol use during pregnancy have been reported to be much higher among some sub-populations. For example, among pregnant Inuit women in northern Quebec (Canada), alcohol use was reported to be 60.5%, which is more than ten times higher than the estimate (10%) for the general population of pregnant women in Canada [25].

## 2. Effects of Alcohol Use on Fetus

Alcohol is a teratogen that can readily cross the placenta, resulting in damage to the developing embryo and fetus. Studies (both animal and clinical) have demonstrated that ethanol diffuses through the placenta and distributes rapidly into the fetal compartment, accumulating in the amniotic fluid [26]. This reservoir causes greater fetal exposure to ethanol and is intensified by fetal swallowing, caused by the fetal kidneys excreting xenobiotics into the amniotic fluid, which is then swallowed by the fetus [27]. Alcohol has a prolonged effect on the fetus due to amniotic accumulation, reduced concentrations of fetal metabolic enzymes, and reduced elimination, which results in damage to the developing embryo and fetus.

The identification of the range of adverse outcomes from prenatal exposure to alcohol is still an emerging field. Thus far, fetal exposure to alcohol is an established risk factor for a number of adverse outcomes, including stillbirth [28], spontaneous abortion [29], premature birth [30,31,32], intrauterine growth retardation [32,33], low birthweight [32,34] and FASD [35].

Cellular responses resulting from prenatal exposure to the teratogen alcohol is an area of considerable interest to a wide range of researchers and clinicians. Currently, alcohol is known to be teratogenic for all organ systems of the developing fetus [36]. Alcohol seems to be especially harmful to the developing nervous system. Some of these mechanisms, include increased oxidative stress to the central nervous system (CNS) [37], impaired angiogenesis and neurogenesis that occurs during brain development in utero [38], increased cell death in various brain structures [39], as well as disruptions to the endocrine system [40], to gene expressions [41] and to prostaglandin synthesis [42]. Prenatal alcohol exposure dysregulates amino acid homeostasis and induces excitatory neurotoxicity, increasing taurine levels as a neuroprotective response [43].

PAE is generally associated with reduced overall brain volume in exposed individuals, which can be observed at birth and later in life. Specifically, individuals with PAE may have reduced gray and white matter in the cerebrum and cerebellum, and reduced gray matter in brain structures including the amygdala, hippocampus, putamen, caudate, thalamus and pallidum [44]. Poorer cognitive and behavioural outcomes are associated with larger brain volume decreases from PAE; however, rates of brain volume growth are similar in early childhood and there are abnormal brain-behaviour connections displayed in children with PAE, suggesting there is a well-defined, long window of opportunity for intervention [45]. The asymmetry of the cerebral cortex thickness throughout development is preserved in individuals with PAE despite their thinner cortex and reduced globally reduced volume, suggesting that typical brain developmental patterns do occur in individuals with PAE and that deficits in cognitive performance are more related to the teratogenic exposure in utero [44].

In addition to the substantial cognitive, neurological, and behavioural deficits resulting from damage to the central nervous system, PAE can also lead to organ defects found in the liver, kidney, and heart, as well as disruptions in the gastrointestinal and endocrine systems [46,47]. Consistent and high levels of alcohol intake on a daily basis during pregnancy are associated with significant fetal impairments in gross and fine motor function observed in childhood [48]. It is theorized that PAE programs the fetal HPA axis to have increased tone, affecting stress response and regulation during baseline and stressful conditions throughout life [40]. This increased tone may be linked to increased reactivity in adverse life events and may represent an increased vulnerability to depression, as HPA dysregulation is commonly found in individuals with depression [49]. There is also preliminary evidence to suggest that PAE can adversely impact immune function later in life as a result of changes in cytokines and lymphocytes that are related to atopic allergy and infection outcomes [50]. While more research is needed in this area, this early finding is important given the relationship between stress reactivity and immune function, suggesting that individuals with PAE are even more susceptible to various comorbidities.

Outcomes from alcohol exposure are modified by a large number of effect modifiers, which include timing and amount of alcohol consumption (dose-dependent), maternal/fetal polysubstance exposure (smoking, other drugs, prescription medications (both prescribed and diverted), nutritional deficiencies, exposure to environmental stressors (e.g., domestic violence, poverty, homelessness), comorbid mental disorders (e.g., depression, anxiety), and peer pressure from friends, families and especially partners’ substance use patterns during pregnancy [51,52,53]. Some patterns of co-exposure are observed to be more detrimental, especially drinking alcohol and nicotine or cannabis exposure [54,55,56]. It has been shown that prenatal exposures to alcohol and nicotine are more potent influences on developing fetal brain structures than cocaine [57].

A growing body of evidence supports epigenetic etiology in the development of FASD due to ethanol-related changes in gene expression (without changes in the DNA sequence), particularly from 3 to 8 weeks of gestation [58]. The timing and pattern of prenatal alcohol exposure affects the alcohol-induced alterations; for example, binge drinking in the first trimester can affect cell proliferation, whereas cell migration/differentiation is affected in the second, and cellular networking is affected by binges in the third trimester [59]. Exposure to alcohol can affect biochemical reactions in the methionine–homocysteine cycle which is linked to epigenetic changes including abnormal DNA methylation [58] and induces oxidative stress which leads to DNA damage [60] affecting a fetus’s cell proliferation and differentiation. Interestingly, chronic paternal alcohol use is associated with epigenetic dysregulation in neonates as well [61]. Furthermore, rodent studies have found that prenatal alcohol exposure affects gene transcription in adolescence [62].

### 2.1. Fetal Alcohol Spectrum Disorder

The most dramatic manifestation of PAE is Fetal Alcohol Spectrum Disorder (FASD). FASD describes a wide range of effects, these are broadly clustered into three categories: (1) cognitive impairments including central nervous system damage, congenital anomalies, prenatal or postnatal growth restrictions; (2) characteristic dysmorphic facial features; and (3) deficits in cognitive, behavioral, emotional, and adaptive functioning. Since the first description of FAS by Jones and Smith in 1973 [63,64], the terminology and the diagnostic guidelines have changed numerous times. Depending on the classification system, FASD includes several alcohol-related diagnoses, such as Fetal Alcohol Syndrome (FAS), partial FAS (pFAS), alcohol-related neurodevelopmental disorder (ARND), and Alcohol-Related Birth Defects (ARBD) [65,66]. The Canadian framework utilizes a single designation of FASD as a diagnostic term, with the specification of the presence or absence of the characteristic sentinel facial features [67]. In addition, the category of neurodevelopmental disorder associated with prenatal alcohol exposure (ND-PAE) is included in the Diagnostic and Statistical Manual of Mental Disorders, Fifth Edition (DSM-5; [68]). Although the criteria for FASD diagnoses have been described thoroughly, the diagnosis of FASD remains challenging, and the specific assessment techniques used to make the definitive diagnosis are still debated, especially for ARND. It is important to note that FASD captures only a modest proportion of damage to the developing fetus from alcohol exposure, so not every child with PAE will be diagnosed with FASD [22].

The risk of developing FASD is related to the patterns (the quantity, frequency, duration) of alcohol consumption, as well as other factors. It has been well documented in both animal and human studies that larger quantities of PAE and sustained drinking throughout all trimesters of pregnancy result in greater physical and cognitive deficits, and the appearance of facial features in FAS [69]. Binge drinking is associated with the highest risk of fetal damage [52]. However, many studies show that low and moderate drinking during pregnancy is also associated with fetal damage [20,21].

Research studies show that many other risk factors can also influence development of FASD, which include a smaller body profile of mother (height, weight, and body mass index [BMI]), maternal age, low socioeconomic status and smoking [52], a poor nutritional status (deficit of riboflavin, calcium, and zinc; [70], genetic polymorphisms [71,72], and paternal alcohol consumption [73].

Several categories of biomarkers of PAE and FASD have been proposed. These include biomarkers demonstrating adverse outcomes for Fetal Alcohol Syndrome including anatomical markers (e.g., facial abnormalities; micrognathia); biomarkers of neurological impairments (e.g., reduced memory retention), developmental biomarkers (e.g., delayed motor learning) and neurobehavioral biomarkers (e.g., deficits in executive functioning) [74]. For example, lower serum concentrations of insulin growth factors IGF-I and IGF-II have been found to be a reliable biomarker of prenatal alcohol exposure in children [75]. Certain imaging studies are proposed to be biomarkers for brain differences associated with FASD, including the use of computerized tomography, magnetic resonance imaging and positron emission tomography [74]. In addition, a number of biomarkers for PAE present at birth are available based on meconium and cord blood [74,76,77].

FASD has a very broad phenotype and is further complicated by high rates of comorbidity. More than 400 disease conditions, spanning across 18 of 22 chapters of the ICD-10 have been reported to co-occur in people diagnosed with FASD [36]. The most prevalent conditions that occur in individuals with FASD are found within the International Statistical Classification of Diseases (ICD-10) chapters of “Congenital malformations, deformities, and chromosomal abnormalities” (43%) and “Mental and behavioral disorders” (18%) [78]. Some comorbid conditions that are highly prevalent among people with FASD include abnormal results of function studies of the peripheral nervous system and special senses, conduct disorder, chronic serous otitis media, receptive and expressive language disorders, and visual, developmental, cognitive, mental, and behavioral impairments, with prevalence estimates of these conditions ranging from 50% to 91% [36]. FASD, as indicated by the sheer number of conditions found to co-occur in this population, is a multifaceted spectrum of disorders, affecting multiple organs and systems.

The complexity and chronicity of FASD affects both the affected individual and their family. In many cases, people with FASD require lifelong support from a wide range of support services, including health care, social assistance, and remedial education. Accordingly, it has been shown that FASD has a substantial economic impact on any society [79,80,81]. In Canada, the annual cost attributable to FASD was estimated to be between CAD1.3 billion and CAD2.3 billion [81]. In North America, the lifetime cost for a complex case of FASD has been estimated to be more than CAD1 million [82]. Individuals from all socio-economic and ethnic backgrounds are affected by FASD [83]. The deficits expressed by individuals with FASD vary broadly in severity and type. Moreover, FASD is intergenerational and familial. Children with an affected sibling are at a higher risk of having FASD [84].

### 2.2. Changes over the Lifespan of People with Fetal Alcohol Spectrum Disorder

The neurodevelopmental impairments associated with PAE resulting in FASD lead to other disabilities later in life. These disabilities include, but are not limited to, academic failure, substance abuse, mental health problems, frequent contact with law enforcement, and inability to live independently and obtain and/or maintain employment—all of which have lifelong implications [69]. These generally are referred to as secondary disabilities, which increase the demands and costs on existing service systems. This includes the health care system with increased demand for services [85], educational systems with need for special services for these children and adolescents, corrections systems beginning in early adolescence and extending across adult life, mental health systems across the lifespan, social services and child protection services and developmental disabilities.

### 2.3. Prevalence of Fetal Alcohol Spectrum Disorder

Globally, the prevalence rates of FASD among the general population were estimated to be 77.3 per 10,000 people [22,86].The prevalence of FASD in the general population was estimated to be highest in the European Region, at 198.2 per 10,000 people, and lowest in the Eastern Mediterranean Region, at 1.3 per 10,000 people (Table 2; [22,86]), respectively. These estimates are in line with the above-reported prevalence of alcohol use during pregnancy. The five countries with highest prevalence of FASD per 1,000 were South Africa (111.1), Croatia (53.3), Ireland (47.5), Italy (45.0), and Belarus (36.6) [86]. The five countries with the lowest prevalence of FASD (<0.1 per 1,000) were Oman, United Arab Emirates, Saudi Arabia, Qatar, and Kuwait [86].

Recent multi-site active case ascertainment studies in the United States have estimated the prevalence of FASD to be 5% among students in grade one [21,87,88,89]. In Canada, the population-based prevalence of FASD among elementary school children was estimated to be between 2% and 3% [90]. These studies found that nearly all of these students in the United States and all of these students in Canada had not been previously diagnosed with FASD.

The prevalence of FASD in the Western Cape Province of South Africa, a region known for wine production and a high prevalence of binge drinking among women of childbearing age, has been reported to be 135.1 to 207.5 per 1000 (13.5–20.8%) among first grade students—one of the highest FASD prevalence rates in the world [91].

A systematic review and meta-analysis revealed that FASD prevalence is much higher in certain sub-populations such as children in care, correctional, or special education programs, specialized clinical populations, and Aboriginal populations, compared to the general population [83]. For example, compared to the general population, the prevalence of FASD among children in care was 32 times higher in the United States and 40 times higher in Chile, the prevalence of FASD among adults in the Canadian correctional system was 19 times higher, and the prevalence of FASD among special education populations in Chile was over 10 times higher [83].

FASD prevalence rates reported in individual studies are extremely alarming. For instance, the prevalence of FASD was 62% among children with intellectual disabilities in care in Chile [92], over 52% among adoptees from Eastern Europe [93], and approximately 40% among children residing in orphanages in Lithuania [94]. The highest prevalence estimates of Fetal Alcohol Syndrome (FAS), which ranged between 46% and 68%, were reported in Russian orphanages for children with developmental abnormalities [95]. Additionally, the prevalence of FASD among youth in correctional services was over 23% in Canada [96], and over 14% among psychiatric care populations in the United States [97].

These findings demonstrate the large service and cost burdens of FASD across various systems of care and reflect a substantial global health problem [81]. It is important to provide caregiver education, diagnosis-informed interventions, lifelong services and long-term risk reduction to individuals with FASD to reduce the prevalence of secondary disabilities. Appropriate interventions, as well as diagnostic and support services, must be available to individuals with FASD from an early age in order to decrease their chances of becoming involved with the legal system, whether as a victim or as an offender. FASD is a huge risk factor for incarceration. It was estimated based on available findings that, on any given day in a specific year, children and adolescents with FASD are 19 times more likely to be incarcerated compared to children and adolescents without FASD [98]. Lastly, high prevalence rates of FASD among special education and specialized clinical populations are not surprising given that individuals with FASD are at an increased risk of having learning difficulties and mental health problems, and of experiencing developmental delays [36].

### 2.4. Fetal Alcohol Spectrum Disorder: Global Implications

Globally, there are 130 million live births each year. In 2016, 32.1% of women of childbearing age globally consumed alcohol in the past year [4] and 10% of women consumed alcohol during pregnancy [22]. These data demonstrate that every year, 13,000,000 pregnancies are alcohol-exposed in the world. Based on a crude estimate of exposure outcomes from a meta-analysis, an estimated one in every 13 alcohol-exposed pregnancies will result in FASD in one specific year [24]. This broad estimate suggests that out of the 13 million alcohol-exposed pregnancies, 1 million of these pregnancies will result in a baby born with FASD. To put this in perspective, this results in 2739 new cases of FASD each day, or 114 every hour, or 2 per minute. The cumulative impact is astonishing. Globally, the number of children with FASD aged from birth through 18 years of age is 18,000,000. We have previously noted that only a small number of individuals with FASD have been diagnosed: perhaps only one of every 800 to 1000 cases [99]. FASD is especially underdiagnosed in certain age groups, as tens of millions of adults and elderly people remain undiagnosed. 

These are conservative estimates. Since a large portion of pregnancies globally are unplanned (44%) [100], many of the 32% of women of childbearing age who already consume alcohol are at risk of having an alcohol-exposed pregnancy in the early stages, before pregnancy recognition typically occurs. Alcohol use during pregnancy is also widely underreported [101,102] so the number of pregnancies with prenatal alcohol exposure (PAE) could be much higher. This would also increase the estimated number of births of children with FASD.

In addition, the number of comorbid disorders found to co-occur in individuals with FASD may also account for the lower prevalence estimates of FASD (i.e., underdiagnoses), likely due to the shadowing that might occur by the other disease conditions. Often, clinicians diagnose the immediate condition that has brought the individual in to seek medical treatment (e.g., problems with concentration), rather than taking into consideration the potential associations and underlying causes of the condition or illness (e.g., PAE).

### 2.5. Fetal Alcohol Spectrum Disorder and Mortality

One of the main neglected manifestations of FASD is premature mortality. The majority of people who may have met the diagnostic criteria for FASD die before they get a chance to be diagnosed. Deaths from stillbirth, sudden infant death syndrome and infectious illness are examples [103,104,105].

People diagnosed with FASD have a five-fold increase in risk for a premature death compared to people without FASD [105]. Since this risk is familial, their siblings similarly have a higher risk of mortality, irrespective of FASD diagnosis [106]. Having children with FASD is an important risk factor in maternal mortality [107,108]. In one case–control study in North Dakota (United States), mothers of children with FASD had a 44.82-fold increase in mortality risk compared to control mothers of the same age [108]. Among the deaths of birth mothers of children with FASD, 87% were among women under the age of 50 years, and among the causes of death for these cases, 67% was attributed to cancer, accidents, or classified as alcohol-related [108].

### 2.6. Fetal Alcohol Spectrum Disorder and Parenting

The complexity of parenting is increased for parents of children with FASD due to multiple issues, including sleep and eating disorders, fine and gross motor delays, toileting, speech and language disorders, need for medication(s), ongoing travel (often years) for therapies, learning and behavior impairments at school, and unavailability of childcare for children with complex behavioral impairments as well as of respite care services [109,110,111,112]. The complexity of parenting increases during adolescence, as young adult life with FASD may be especially complex. Early recognition of FASD and early emphasis on prevention of these problems and secondary disabilities are seen to be most useful in decreasing demands on parents [109].

## 3. Treatment Options

To date, there is no known treatment to reverse alcohol-induced damage to the fetus; however, many animal and human research studies have explored treatment options to reverse or prevent the mechanisms of alcohol teratogenicity on fetus [113]. For instance, animal studies have demonstrated ethanol-induced oxidative stress reduction by administering vitamins C and E [114,115,116,117], astaxanthin [117,118], curcumin [119,120] and resveratrol [121,122]. Doses of Vitamin E were found to reduce and even prevent Pukinje cell loss in rats [123,124], and when paired with beta carotene, doses of Vitamin C can also reduce or ameliorate hippocampal cell loss [124,125]. In zebrafish embryos, doses of Vitamin A were found to reverse small eye and body length defects, but were unable to reverse heart edema and other physical anomalies following PAE [124,126,127]. It was also shown that zinc can decrease physical abnormalities when administered at the time of ethanol exposure in mice [124,128,129]. Administering food supplements with folic acid, selenium, DHA, L-glutamine, boricacid, and choline [124,130] to pregnant women with risk of developing a child with FASD, also demonstrated that it can reduce the severity of ethanol-induced toxicity [124,131]. Recent studies show that low maternal iron reserves during pregnancy are associated with more prevalent FASD features where there is PAE; this is especially important as women of reproductive age who are heavy users of alcohol tend to have low iron stores [132]. Furthermore, while there is a breadth of evidence using animal models, clinical trials are needed to study interventions and supplementation options for humans [117].

## 4. Limitations

The current review has several strengths, including an overview of the most up-to-date epidemiological data on alcohol use in pregnancy and FASD in conjunction with what is known about the effects of alcohol on the fetus, including individual and population-level impacts. These data must be understood in light of several existing limitations, however. First and foremost, alcohol use during pregnancy is underreported and in addition, FASD is largely undiagnosed. Some countries may not have available data on alcohol use during pregnancy (e.g., countries not included in WHO Member States) and though the global estimate is 10%, this varies widely across countries. Furthermore, studies on maternal alcohol consumption are often not based on representative sampling strategies, and often do not include data on alcohol use patterns, pregnancy planning, or timing of pregnancy recognition. The current review presents meta-analytic estimates of FASD prevalence in general and sub-populations based on active case ascertainment (ACA) studies, which are the gold standard; however, case definitions and diagnostic criteria for FASD vary widely across studies and in different countries.

## 5. Conclusions

The detrimental effects of alcohol on a fetus leading to preventable chronic disabilities should be recognized and addressed as a global public health issue. The increasing incidence and prevalence of PAE and FASD at alarming rates is a public health exigency which demands strategic and timely interventions for both pregnant and childbearing-aged women who consume alcohol, and for their offspring who may be at risk of PAE. Prevention initiatives aimed at reducing alcohol use prior to and during pregnancy should be implemented worldwide.

There must be recognition that FASD is not restricted solely to disadvantaged groups, but rather that it may occur throughout society, regardless of socio-economic status, education, or ethnicity [90]. Therefore, efforts must be made to better educate the general population (adult women and men, children, and teenagers) about the risks of alcohol use (especially binge drinking and frequent drinking) during pregnancy.

Appropriate screening for alcohol use in all women of childbearing age, in combination with health promotion before conception, contraceptive counselling, brief interventions, and referrals to substance abuse programs where appropriate, should become a routine standard of care. Research has shown that easy access to substance use programs utilizing effective treatment of identified cases of alcohol dependence or AUDs among pregnant women could reduce the risk of PAE and having children with FASD [133,134]. Collaborative research projects and maternal health promotion programs (e.g., the Women-Infant-Child (WIC) program or the Parent Child Assistance Program (PCAP)) can be funded to help identify mothers who are drinking during pregnancy and therefore infants and young children with PAE who are at risk for FASD. Such programs have been shown to be cost-effective, and similarly, it has been estimated that preventing one case of FASD incurs only 3% of the costs it would require to provide support services to individuals with FASD [135].

Screening for PAE could lead to close monitoring of a child’s development, facilitate early diagnosis, and the implementation of timely interventions, if necessary. It would also prevent the occurrence of secondary disabilities later in life and the occurrence and/or recurrence of prenatal and postnatal alcohol exposure within families at risk for FASD. Improved access to FASD diagnosis also is an opportunity to identify mothers who not only have maternal risk factors for FASD but also have children with FASD. This is a priority population for the prevention of alcohol exposure in future pregnancies. Among people diagnosed with FASD, specific efforts must be made to prevent school failure, victimization, incarceration, and the development of substance use disorders. Effective and consistent execution of universal screening can assist all countries in the development of surveillance systems to monitor the prevalence of alcohol use during pregnancy and of FASD, over time, in order to identify vulnerable populations, develop resources, and evaluate the effectiveness of prevention strategies.

To conclude, the negative health outcomes of alcohol exposure on the fetus, including FASD, are preventable and the most effective measure is to completely avoid consuming any type of alcohol during the entire pregnancy and while trying to become pregnant. All countries should implement effective and cost-effective population-based policy options that aim to reduce alcohol use among populations, including women of childbearing age and pregnant women [136,137], which ultimately will decrease the incidence of FASD and other negative health outcomes of children and their mothers.

## Figures and Tables

**Table 1:** Adult per capita consumption and the prevalence of current drinking, former drinking, lifetime abstention, and heavy episodic drinking among women of childbearing age (15 to 49 years of age) in 2016.

Regions	Per Capita Consumption (Litres)	Prevalence (%)			
		Current Drinking	Former Drinking	Lifetime Abstention	Heavy Episodic Drinking
Global	2.3	32.1	56.7	11.3	8.7
WHO regions					
African	2.1	21.7	69.4	9.0	7.6
America	3.1	42.9	25.0	32.0	10.5
Eastern Mediterranean	0.1	1.3	97.8	0.9	0.1
European	4.6	53.9	30.3	15.9	18.7
South-East Asia	1.4	22.5	70.5	7.0	5.2
Western Pacific	2.8	42.7	49.6	7.6	10.7
World Bank regions					
Low-income economies	1.2	17.4	73.8	8.8	5.5
Lower-middle-income economies	1.5	20.6	71.3	8.1	5.0
Upper-middle-income economies	2.7	37.6	50.7	11.8	10.4
High-income economies	4.5	60.7	18.4	20.9	17.3

Source: The Global Information System on Alcohol and Health [12].

**Table 2:** Global prevalence of any amount of alcohol use and binge drinking (4 or more drinks on a single occasion) during pregnancy, and of FAS and FASD among the general population, by WHO Region in 2012, and corresponding 95% confidence intervals.

Region	Alcohol Use (Any Amount) during Pregnancy (%) ^a^	Binge Drinking during Pregnancy (%) ^b^	FAS (per 10,000)	FASD (per 10,000) ^c^
Globally	9.8 (8.9, 11.1)	-	9.4 (9.4, 23.3)	77.3 (49.0, 116.1)
WHO Regions				
African	10.0 (8.5, 11.8)	0.1 (0.1, 6.1)	8.9 (8.9, 21.5)	78.3 (53.6, 107.1)
America	11.2 (9.4, 12.6)	0.1 (0.1, 5.6)	11.0 (11.0, 24.0)	87.9 (63.7, 132.4)
Eastern Mediterranean	0.2 (0.1, 0.9)	-	0.2 (0.2, 0.9)	1.3 (0.9, 4.5)
European	25.2 (21.6, 29.6)	0.0 (0.0, 5.3)	24.7 (24.7, 54.2)	198.2 (140.9, 280.0)
South-East Asia	1.8 (0.9, 5.1)	-	1.3 (1.3, 8.1)	14.1 (6.4, 53.1)
Western Pacific	8.6 (4.5, 11.6)	0.0 (0.0, 3.5)	7.7 (7.7, 19.4)	67.4 (45.4, 116.6)

Source: Popova et al., 2017 [23]. ^a^ The prevalence of any amount of alcohol use during pregnancy is inclusive of the prevalence of binge drinking during pregnancy. ^b^ It was not possible to estimate the prevalence of binge drinking during pregnancy for the Eastern-Mediterranean or South-East Asia Regions due to a lack of available data for countries in these regions; therefore, the global prevalence could not be estimated. ^c^ The prevalence of FASD includes the prevalence of FAS. © Canadian Science Publishing.

**Table 3:** Estimated annual number of cases of FASD in the five countries (Ireland, Belarus, Denmark, the United Kingdom, and Russia) reporting the highest prevalence of alcohol use during pregnancy in the world in 2016.

Country	Estimated Number of Cases with PAE (%) *	Annual Births (Prenatally Alcohol Exposed Births)	Annual Number of Cases with FASD *	Number of Cases with FASD from Birth up to Age 18
Ireland	60.4	55,959 (33,799)	2599	46,782
Belarus	46.6	107,930 (50,295)	3868	69,624
Denmark	45.8	61,167 (28,014)	2154	38,772
United Kingdom	41.3	640,370 (264,472)	20,344	366,192
Russia	36.5	1,440,000 (525,600)	40,430	727,775
Total cases with FASD		2,305,426 (902,180)	69,395	1,249,110

* Based on the assumption that one in every 13 alcohol-exposed pregnancies will result in FASD in any given year [22,23,24].
